# Germination characteristics associated with nicosulfuron resistance in *Amaranthus retroflexus* L.

**DOI:** 10.1371/journal.pone.0308024

**Published:** 2024-08-12

**Authors:** Yingying Zhang, Xian Xu, Bochui Zhao, Binghua Li, Zhizun Qi, Yu Wang, Guiqi Wang, Yaofa Li, Zhaofeng Huang, Xiaomin Liu

**Affiliations:** 1 Key Laboratory of Crop Cultivation Physiology and Green Production of Hebei Province, Institute of Cereal and Oil Crops, Hebei Academy of Agriculture and Forestry Sciences, Shijiazhuang, China; 2 College of Life Sciences, Hebei University, Baoding, China; 3 Institute of plant protection, Heilongjiang Academy of Agricultural Sciences, Harbin, China; 4 Plant Protection Institute, Hebei Academy of Agricultural and Forestry Sciences, Baoding, China; 5 State Key Laboratory for Biology of Plant Diseases and Insect Pests, Institute of Plant Protection, Chinese Academy of Agricultural Sciences, Beijing, China; Canakkale Onsekiz Mart University, TÜRKIYE

## Abstract

Nicosulfuron-resistant biotype (R) and -sensitive biotype (S) *Amaranthus retroflexus* L. seeds were subjected to different temperature, light, salt, osmotic potential, pH value and burial depth treatments. The difference in germination response of two populations to the above abiotic environmental factors was used to study the fitness cost of nicosulfuron-resistance evolution in *A*. *retroflexus*. The aim is to find a powerful tool for weed control in the presence of evolutionary resistance selection. The results of this experiment showed that the germination rate and germination index in S population were generally higher than that in R population. When the salt stress was 80 mM, the water potential was -0.1 Mpa ~ -0.4 Mpa, and under strong acid and alkali conditions, the germination index in S population was prominently higher than that in R population (*p*<0.05). The delayed seed germination in R population indicated that its nicosulfuron resistance may be linked to seed biochemical compositions that altered seed germination dynamics. The resistant and sensitive biotype of *A*. *retroflexus* had differently favourable adaptability in diverse environments. Salt, osmotic potential and pH value are not the major constraints for *A*. *retroflexus* germination, however, *A*. *retroflexus* are strongly responsive to temperature, light and burial depth. Considering that seeds of *A*. *retroflexus* are unable to reach the soil surface beyond the depth of 6 cm, deep inversion tillage before sowing may be an effective and economical weed management tool for the control of nicosulfuron resistant *A*. *retroflexus*.

## Introduction

*Amaranthus retroflexus* L., native to America, is now widely distributed in many cultivation areas of China. It occurs mainly in soybean and corn fields, and also infests wheat, sweet potato, cotton and other fields [1]. Due to its strong adaptability and reproductive ability, *A*. *retroflexus* has become a malignant weed that competes with crops for nutrients and water, resulting in a significant decrease in grain yield and quality of crops [2].

With the introduction of herbicides in the 1940s, which replaced the labor-intensive weed control measures of early agriculture, herbicides have been the most effective control tool for weed management to date. Nicosulfuron, a sulfonylurea herbicide, has been widely used due to its broad weed spectrum, low application amount and high crop safety [3]. However, intense selection pressure from long-term single application results in the evolution of herbicide-resistant weed biotypes, and now resistant *A*. *retroflexus* populations have been reported worldwide [4]. The existence of resistant weeds reduces the efficacy of corresponding herbicides, makes it difficult to select herbicides, and increases herbicide doses. These problems not only increase the cost of weed control and cause crop yield loss, but also aggravate the risk of herbicide damage and environmental pollution. At present, resistant weeds have become a serious problem in weed control.

Fitness is considered to be an ability that helps an organism establish, survive, and reproduce successfully [5]. The effects of resistance evolution involve multiple traits in several processes of plant growth and ultimately change fitness in different ways. Plant functional traits such as survival, growth, and reproduction can be measured at individual level and determine the fitness components of the plant [6]. The fitness cost in young plants dictates whether they have benefits or drawbacks in competitive settings. Therefore, it’s essential to investigate the manifestation of these fitness costs and the alterations in life history linked to characteristics and events in the plant’s early stages [7].

Seed germination which signifies the beginning of life cycle provides opportunity to judge the evolutionary dynamics of seedling competitive ability. Due to the heterogeneity of environmental gradients, seed germination and relative dormancy after shedding are influenced by diverse environmental factors that modify seed physiology and behavior [8, 9]. In the study of fitness cost of resistant populations, the difference in germination characteristics has been reported as one of the components [10]. There was no major difference in seed germination under diverse temperatures, pH ranges, and light conditions. However, this study showed that the seeds tended to respond differently to a gradient of osmotic and salt stress in resistant and susceptible *Polypogon fugax* populations [10]. *Amaranthus powellii* with Trp574Leu acetylhydroxylate synthase (AHAS) mutations appears to have multiple pleiotropic effects on the initial stages of the plant life cycle, while *Polypogon fugax* had a decreased fitness due to its evolved resistance to aryloxyphenoxy propanoate herbicides [11, 12]. Up to now, however, there is still little research on the different germination responses of resistant *A*. *retroflexus* populations under various stress treatments. The purpose of this study is to: (a) explore the impacts of different environmental gradients such as temperature, light, pH, salt stress, osmotic potential energy, and burial depth on the germination of nicosulfuron-resistant and -sensitive *A*. *retroflexus* populations; (b) compare the germination responses of the nicosulfuron-resistant and -sensitive populations to these treatments; (c) search for effective tools to assist resistant weed management.

## Materials and methods

### Plant material

The resistant population and sensitive population of *A*. *retroflexus* seed were collected from Gaocheng District, Shijiazhuang Hebei (114.73°N, 37.95°E) in 2021, and identified by whole plant bioassay in the laboratory of the Institute of Grain and Oil Crops, Hebei Academy of Agriculture and Forestry Sciences. The nicosulfuron ED_50_ of resistant population (R) and sensitive population (S) were 127 g a.i. ha^-1^ and 4.91 g a.i. ha^-1^, correspondingly.

### Methods

All of the following experiment were completed in the laboratory of the Institute of Cereal and Oil Crops, Hebei Academy of Agriculture and Forestry Sciences. General germination test was conducted in a 9 cm petri dish with two layers of qualitative filter paper (from Hangzhou Fuyang North Wood Pulp Paper Co., LTD., China). In order to prevent water depletion, absorbent cotton was placed in the bottom of the petri dish and 15~20 ml distilled water was added. The incubator was set at a suitable temperature of 35°C/30°C, photoperiod was 12L:12D, and optical density was 3000 lux. Fifty seeds were randomly placed in each petri dish and seed germination was identified when the seed radicle was exposed ≥ 2 mm [13]. The test was conducted for 7 days, the stress test was recorded for 15 days, and the number of seed germination was recorded every 24 hours, after which the germinated seeds were removed from the petri dish.

#### Seed germination tests under temperature and light conditions

The impacts of temperature and light on the period of seed germination of two biological types of *A*. *retroflexus* were tested. The temperatures of day/night fluctuation were set as 25°C/20°C, 35°C/30°C, 40°C/35°C. Under the illumination condition, the photoperiod is 12:12 h light/dark, and the light intensity is 3000 lux. Other methods are consistent with the above test scheme. The statistical layout was therefore a 3x2 factorial with three replicates.

#### Seed germination test under salt stress

Sodium chloride (NaCl) was used to test the salt stress of seeds of two biological types of *A*. *retroflexus*. Salt concentrations were set up: 0, 10, 20, 40, 80, and 160 mM, and distilled water (0 mM) was used as the control. Other methods are consistent with the above test scheme.

The percentage of germination values at different salt concentrations were fitted to a functional three-parameter logistic model using SigmaPlot (v.14.0, SigmaPlot Software, Chicago, IL, USA). The model fitted was:

$$G(\%)=\frac{G_{max}}{1+\left(x/x_{50}\right)G_{rate}}$$

In this equation, G represents the total percentage germination (%) at NaCl concentration x, Gmax is the maximum germination (%), x_50_ is the NaCl concentration for 50% inhibition of the maximum germination, and G_rate_ indicates the slope of the equation.

#### Seed germination test under osmotic stress

To test seed germination under osmotic potential stress, a solution with an osmotic potential of 0, -0.1, -0.2, -0.3, -0.4, -0.5 and -0.6 Mpa was prepared by dissolving 0, 62.6, 99.7, 128.9, 153.9, 176.0 and 196.0 g Polyethylene glycol -6000 (PEG-6000) in 1 L deionized water, respectively. Other methods are consistent with the above test scheme.

#### Seed germination test under different pH values

The seeds were individually set in buffered solutions with pH levels ranging from 4 to 10. A 2 mM potassium hydrogen phthalate buffer was adjusted to a pH of 4 using 1 mol⋅L^-1^ HCl. Similarly, a 2 mol⋅L^-1^ MES [2- (N-morpholino) ethanesulfonic acid] solution was modified to reach pH levels of 5 or 6 by adding 1 mol⋅L^-1^ NaOH. A 2 mol⋅L^-1^ HEPES [N- (2-hydroxymethyl) piperazine-N-(2-ethanesulfonic acid)] solution was set to pH 7 or 8 using the same 1 mol⋅L^-1^ NaOH. Solutions with a pH of 9 or 10 were achieved using a 2 mM tricine buffer, again adjusted with 1 mol⋅L^-1^ NaOH. Deionized water with a pH of 6.6 served as a reference. All other growing conditions mirrored the standard germination procedures.

#### Effect of burial depth on seed emergence

The effect of seed burial depth on seedling emergence was studied in the greenhouse, the soil depth was set to 0, 2, 4, 6, 8, and 10 cm, respectively. Twenty seeds of these two biological types were planted in pots with a height of 10 cm and a diameter of 8 cm, and the soil was 3:1 humus and vermiculite mixed. Pots were watered every other day to maintain the soil moisture. The pots were placed randomly in the greenhouse at 35°C/30°C with a 12 h photoperiod. The seedlings were considered to be emerged when the coleoptile was visible above the soil surface. Emergence was counted daily for 30 d until no further emergence was recorded, and the seedlings were removed after the daily counts.

The germination indexes (GI, respectively) were calculated according to the description by the Association of Official Seed Analysis (1990), using the following formula [10]:

$$GI=\frac{No\;of\;germinated}{days\;of\;first\;count}+\cdots +\frac{No\;of\;germinated}{days\;of\;final\;count}$$

### Statistical analysis

The above tests were repeated twice, and three biological replicates were set up for each germination test. The germination data recorded every 24 hours were combined to calculate the germination rate and germination index. The DPS software(v.2020.3.25, China) by Tukey Honestly Significant Difference test (*p*≤0.05) was applied to analyze the significance of the data. Nonlinear regression analysis was conducted by SigmaPlot (v.14.0, SigmaPlot Software, Chicago, IL, USA) and mean comparison was performed using Fisher’s protected LSD test at *p*≤0.05.

## Results

### Effects of temperature and light on seed germination

**Fig 1:**
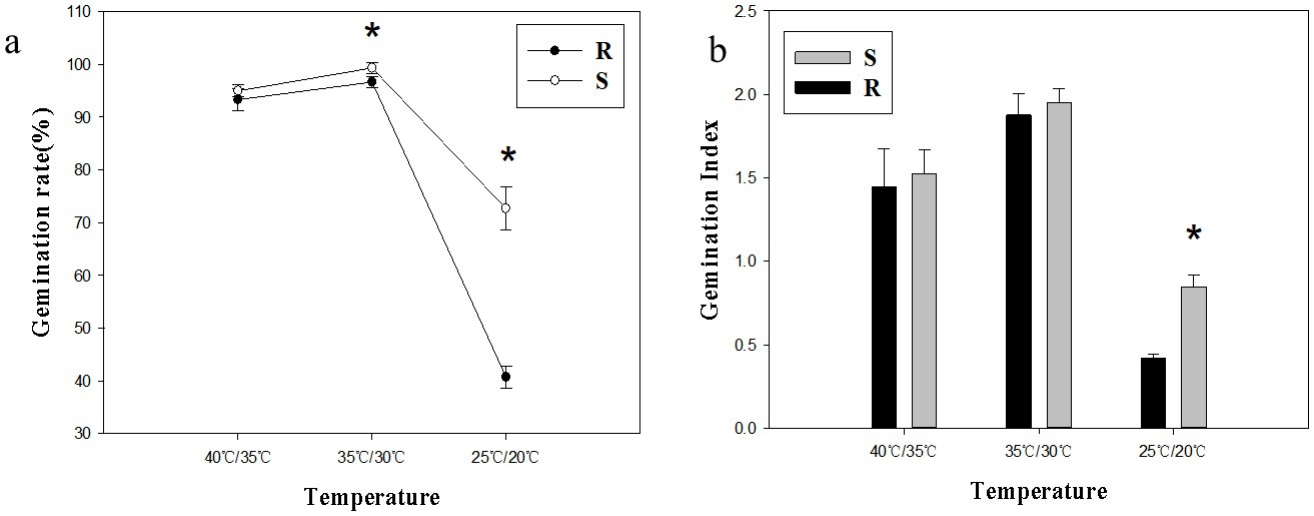
Effects of temperature on seed germination under light conditions. **(a) Germination rate; (b) Germination Index**. Values represent the mean of six replications with 50 seeds per replicate. Error bars represent standard error of the means. The abbreviations R and S represent for the resistant and sensitive *Amaranthus retroflexus* L. populations, respectively. *Indicates significant differences (*p*<0.05) by Tukey’s test.

**Fig 2:**
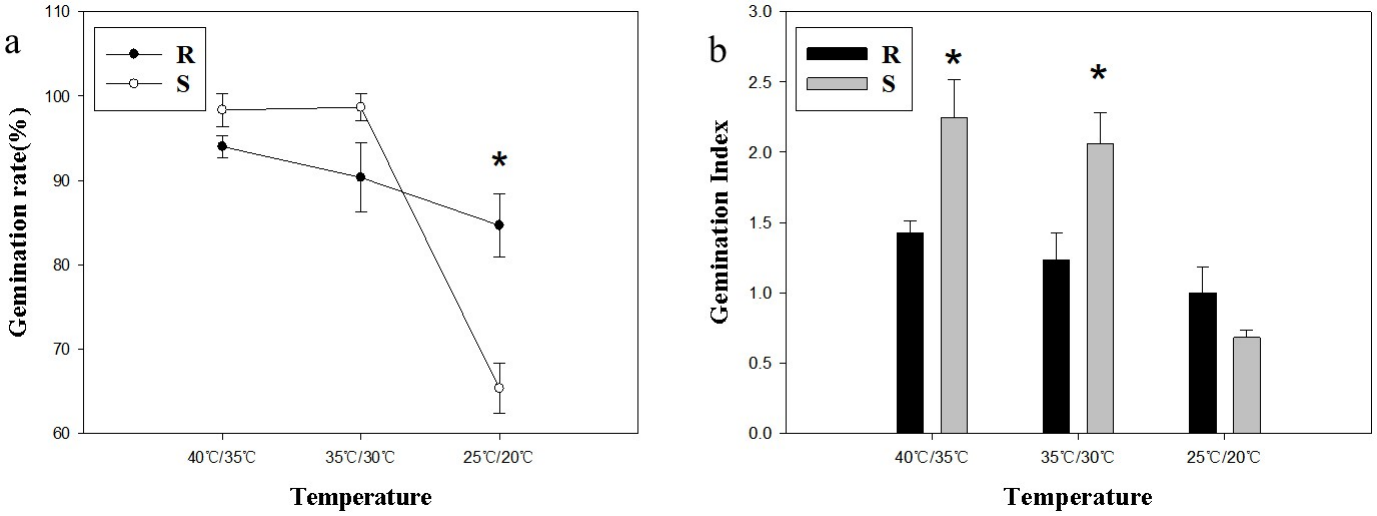
Effects of temperature on seed germination under darkness conditions. **(a) Germination rate; (b) Germination Index**. Values represent the mean of six replications with 50 seeds per replicate. Error bars represent standard error of the means. The abbreviations R and S represent for the resistant and sensitive *Amaranthus retroflexus* L. populations, respectively. *Indicates significant differences (*p*<0.05) by Tukey’s test.

In terms of the germination ability, S population was higher than R population under light conditions. The germination rates of R and S populations were the highest at 35°C/30°C, reaching 96.7% and 99.3%, respectively. At low temperature of 25°C/20°C, the germination rate of *A*. *retroflexus* decreased sharply (Fig 1a). Under darkness, the germination rate of R and S populations ranged from 82.7% to 94% and 65.3% to 98.7% respectively, both of which were lower than those under light condition. At dark and low temperature conditions, the germination rate of resistant population was significantly higher than that of sensitive population (Fig 2a). The germination index of S population was significantly higher than that of R population under the conditions of low temperature with light (*p*<0.05; Fig 1b). However, at low temperatures without light, there was no significant difference between R and S populations, while at higher temperatures, S population had significantly higher germination index (*p*<0.05; Fig 2b).

### Effect of salt stress on seed germination

**Fig 3:**
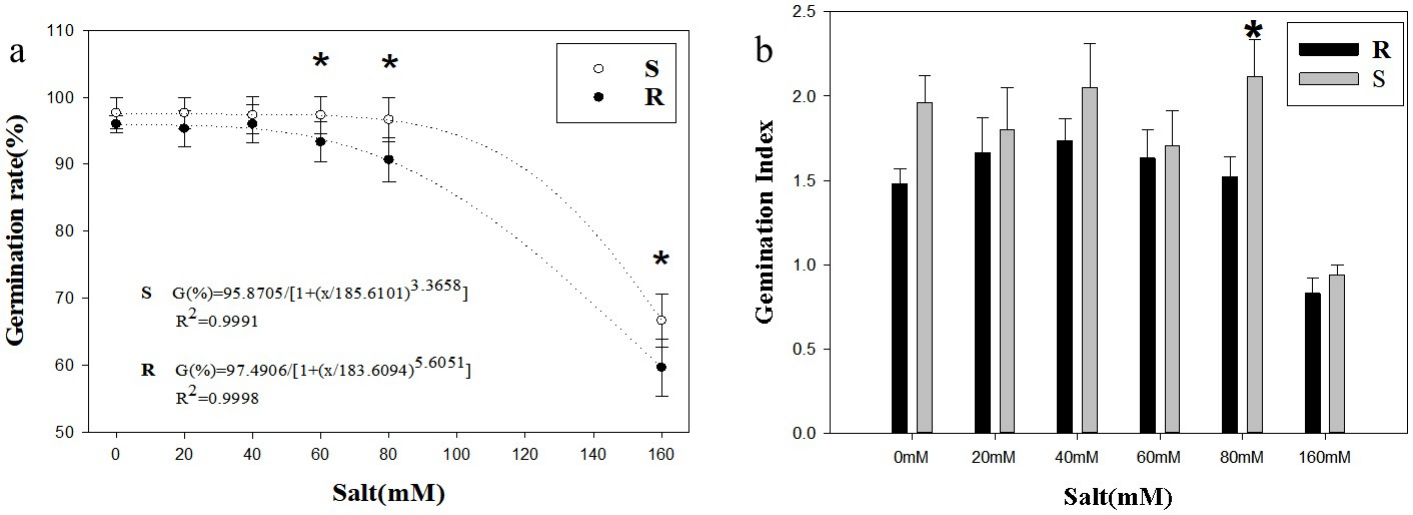
Effect of salt stress on seed germination. **(a) Germination rate; (b) Germination Index**. Values represent the mean of six replications with 50 seeds per replicate. Error bars represent standard error of the means. The abbreviations R and S represent for the resistant and sensitive *Amaranthus retroflexus* L. populations, respectively. *Indicates significant differences (*p*<0.05) by Tukey’s test.

The germination rate of the two populations decreased gradually under the environment in which salt stress was between 0 mM and 80 mM, but both were above 90% (Fig 3a). When salt stress was 60 mM, 80 mM, 160 mM, the germination rate in S population was significantly higher than that in R population (*p*<0.05; Fig 3a). When salt stress was 80 mM, the germination index in S population was significantly higher than that in R population (*p*<0.05; Fig 3b). As salt stress increased to 160 mM, the germination ability in R and S populations dropped sharply to less than 70% (Fig 3a). The overall germination performance in S population was generally higher than that in R population under salt stress (Fig 3a and 3b).

### Effect of osmotic potential stress on seed germination

**Fig 4:**
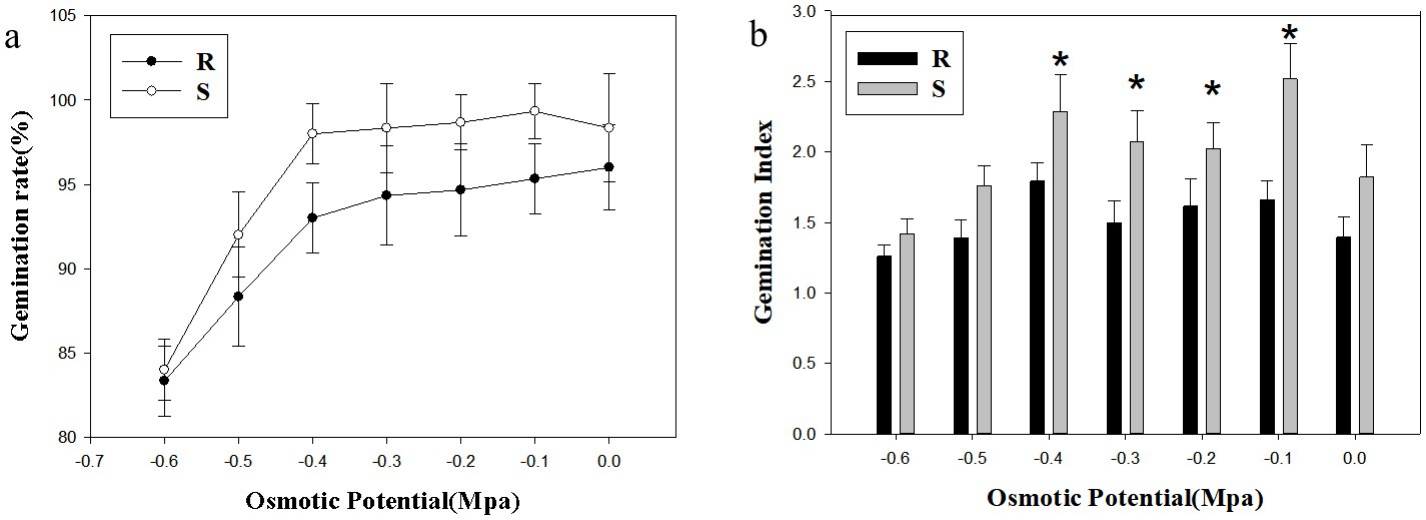
Effect of osmotic potential stress on seed germination. **(a) Germination rate; (b) Germination Index**. Values represent the mean of six replications with 50 seeds per replicate. Error bars represent standard error of the means. The abbreviations R and S represent for the resistant and sensitive *Amaranthus retroflexus* L. populations, respectively. *Indicates significant differences (*p*<0.05) by Tukey’s test.

Under osmotic potential stress treatment, the germination rate of R population was 96%-83.3%, and that of S population was 99.3%-84% (Fig 4a). The germination rate in S population were still higher than that of R population. The germination rate of *A*. *retroflexus* was negatively correlated with water potential stress, and the germination rate of two populations decreased with the increase of osmotic potential. The germination index fluctuated irregularly and was generally higher than 1.0 (Fig 4b). When the water potential was -0.1 Mpa~-0.4 Mpa, the germination index in S population was significantly higher, compared to that in R population (*p*<0.05; Fig 4b).

### Effects of different pH values on seed germination

**Fig 5:**
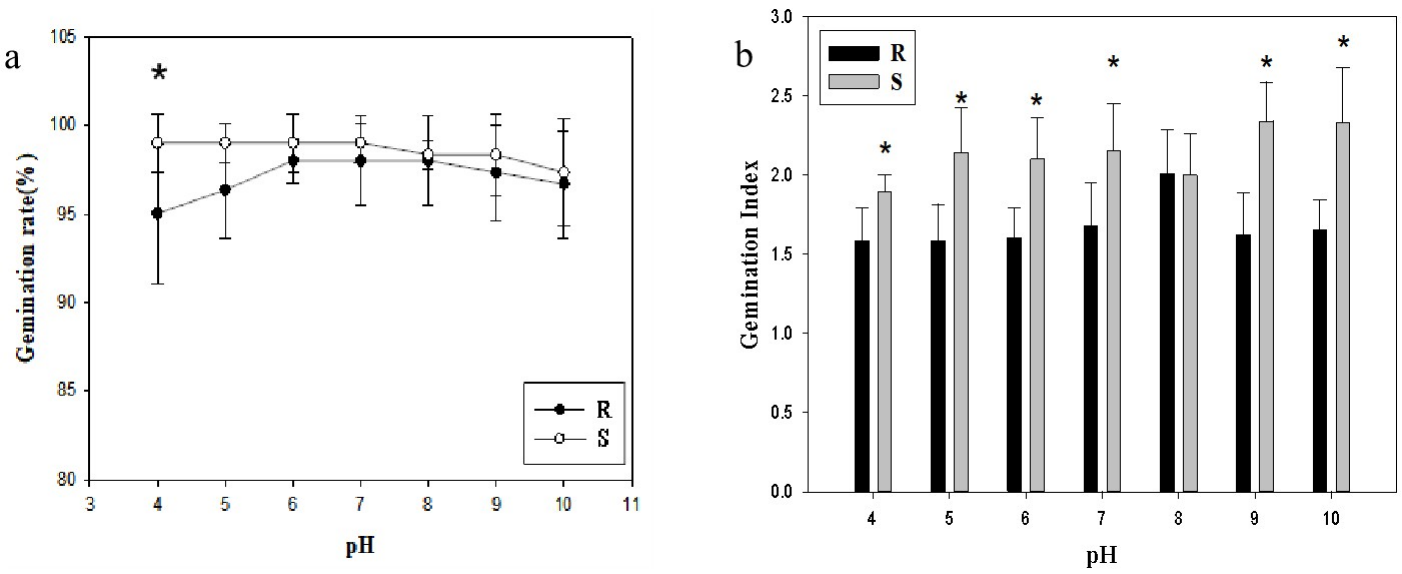
Effects of different pH values on seed germination. **(a) Germination rate; (b) Germination Index**. Values represent the mean of six replications with 50 seeds per replicate. Error bars represent standard error of the means. The abbreviations R and S represent for the resistant and sensitive *Amaranthus retroflexus* L. populations, respectively. *Indicates significant differences (*p*<0.05) by Tukey’s test.

Both the R population and the S population could germinate at all tested pH values, and the R population germinated less than the S population, but the overall germination rate was higher than 95%, and the germination index was higher than 1.5 (Fig 5a and 5b). Under acidic conditions to neutral conditions, the germination rate in S population reached 99%, maintaining a high germination ability. It gradually decreases under alkaline conditions. Compared with the S population, the R population was intolerant to acidic conditions. When pH < 6 or pH > 8, the germination rate in R population gradually decreased, while the germination rate in S population remained at the same ability, reaching 98% under weak acid to weak base environment (Fig 5a). At pH = 4, the germination rate in resistant population was significantly higher than that in sensitive population. When pH = 8, the germination index remained the same in both the R and S populations, but under other pH conditions the R germination index were significantly lower than that in S population (*p*<0.05; Fig 5b).

### Effect of burial depth on seed emergence

**Fig 6:**
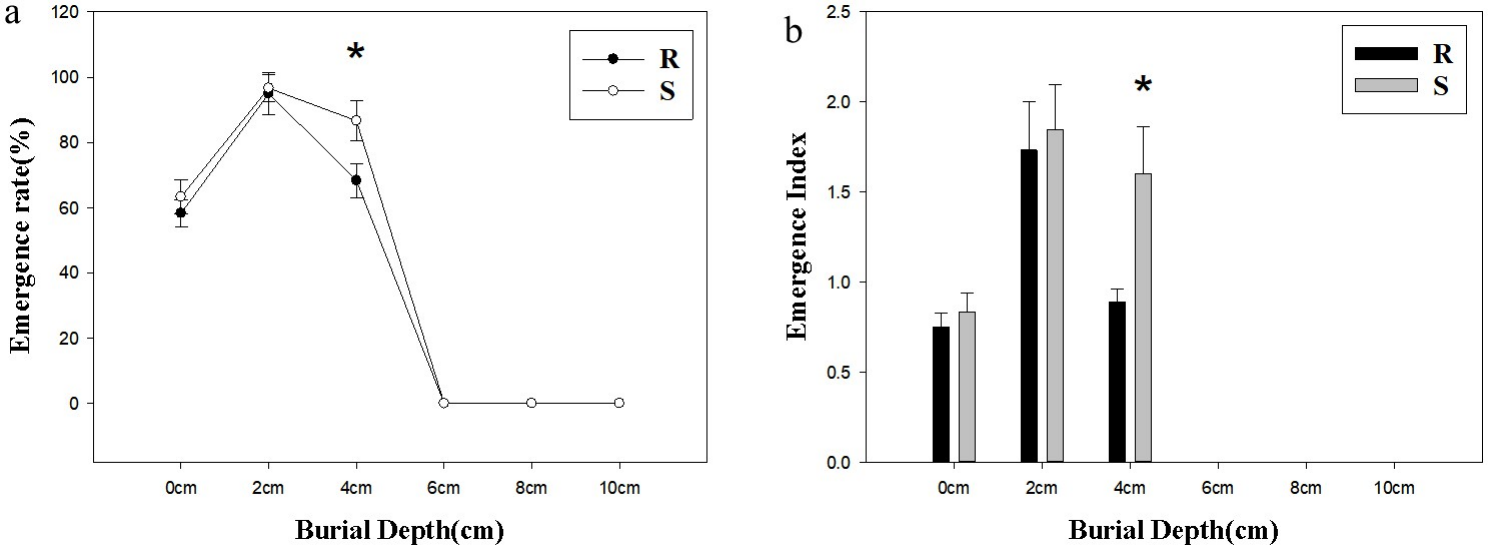
Effect of soil depth on seed emergence of two biological types of *Amaranthus retroflexus* L. (a) Germination rate; (b) Germination Index. Values represent the mean of six replications with 50 seeds per replicate. Error bars represent standard error of the means. The abbreviations R and S represent for the resistant and sensitive *Amaranthus retroflexus* L. populations, respectively. *Indicates significant differences (p<0.05) by Turkey test.

The seeds of *A*. *retroflexus* could emerge at the burial depth of 0~4 cm, but not at the burial depth of 6 cm or deeper. The emergence rate in S population was higher than that in R population, the emergence rate in R population ranging from 58.3% to 95%, and that in S population ranging from 63.3% to 96.7%. The optimal emergence depth of *A*. *retroflexus* was 2 cm, in which depth the emergence rate was the highest (Fig 6a). Emergence index in S population was slightly higher than that in R population at 0 cm and 2 cm, while the emergence rate and the emergence index in S population was significantly higher than that of the R population at 4 cm (*p*<0.05; Fig 6b).

## Discussion

Stress often has negative effects on plants, especially in terms of growth, development and reproduction [14]. It is able to determine the distribution of species, while providing a selective evolutionary pressure on a particular population [15]. As a result of weed resistance evolution, fitness cost can predict the evolutionary trend of resistant and sensitive populations, and seed germination may be the most important phenological event determining the successful establishment of annual plants [16]. High germination ability mean that space is captured more efficiently, thus ensuring their survival and distribution in the population and the preservation of evolutionary traits. This study mainly took into account that *A*. *retroflexus* is an invasive organism with a wide range of habitats, so different stress gradient factors were set up to compare the responses of nicosulfuron-resistant and sensitive populations to temperature, light, salt, pH, osmotic potential and burial depth.

Germination rate of C4 species are reported to increase with temperature. The results of this study are consistant with this characteristic. The experimental data show that the resistant and sensitive populations of *A*. *retroflexus* can germinate in a broad spectrum of temperatures, and the germination rate of them is more than 90% within 24 h under suitable temperature conditions. Light can stimulate the germination of *A*. *retroflexus* to a certain extent. The interaction between temperature and light affected the germination of *A*. *retroflexus*. The germination rate in *A*. *retroflexus* S population was slightly higher than that of R population in the higher temperature, while the difference was not significant, which was consistent with previous study [17]. Despite the difference being relatively small, even a small increase in germination rates could provide a competitive advantage, especially for invasive species [18].

In darkness, the germination rate in R population decreased linearly with the decrease of temperature, and the germination index in S population was significantly higher than that in R population at temperature of 40/35°C and 35/30°C, indicating that the seed vitality of R population was worse than that of S population. At low temperatures, the catabolism of stored proteins slows down and the available levels of free amino acids are reduced [5]. The germination index in R population was higher than that in S population under the relatively low temperature of 25°C/20°C. A similar situation has been found in *Lactuca sativa* L. and *Kochia scoparia* when temperatures are low in late autumn or winter [5, 19]. It was suggested that the rapid germination of R populations at low temperature was related to the increase of the level of branched-chain amino acids [18, 20]. In this study, the minimum temperature was set at 20°C, and this characteristic may be more significant at lower temperatures.

Extreme salinity and osmotic potential levels are usually the main abiotic limiting factors for plants and are not conducive to the germination of many weeds [21–24]. Sodium content modifies soil composition and replaces calcium and magnesium through an anionic exchange process, resulting in water and nutrient deficiencies [25]. It was found that the R and S populations had a certain tolerance to sodium chloride, and the germination rate at 160 mM was still higher than 50%. Although osmotic potential stress increased, the germination rates in R and S populations also gradually decreased, the germination rates remained higher than 80% from 0 Mpa to -0.6 Mpa, indicating that osmotic potential and salt stress were not the main factors affecting the germination of *A*. *retroflexus*.

Under the salt stress and osmotic potential influences, the germination rate in S population was higher than in R population. Similar results were found in other studies for *Polypogon fugax*, *Alopecurus japonicus* [12, 26]. However, for glyphosate-resistant and -susceptible Italian ryegrass, the germination ability of resistant population was reported to be higher than that of S population [27]. When salt stress was 80 mM and water potential was -0.1 Mpa~-0.4 Mpa in the current study, the germination index in S population was obviously higher than that in R population. Compared with the S population, R population has a lower germination rate and germination delay under salt stress and osmotic potential stress, which was expected to be enhanced by a higher probability of escaping pre-planting management measures [28–30].

The seeds had good adaptability and maintained a high germination rate in the range of pH 4~10, suggesting that pH value was not a limiting factor for the germination of *A*. *retroflexus*. Similar conclusions were also obtained in studies on R and S *Alopecurus japonicus* biotypes [26]. Many weeds have been studied and found to have wide germination range on pH, such as *Lolium rigidum* [24], *Polypogon fugax* [31] and *Beckmannia syzigachne* [32]. Based on the data from this experiment, it was found that the resistant population evolved a weak sensitivity to acidic environment compared with the sensitive population. In addition, the vitality of resistant seeds under pH conditions was significantly lower than that of sensitive populations except for weak alkali conditions. The germination of early weed seeds could be controlled by preemergence herbicide, while resistant weeds with irregular emergence would be exposed to herbicides with a high probability and have more opportunities for selective evolution [30].

In this study, the optimal burial depth for germination in R and S populations was 2 cm, and the germination rate decreased when depth of burial increased. The same has been reported for some species of weeds, such as *Brunnichia ovata* [21, 33]. Seeds of *A*. *retroflexus* can not germinate with exposure at a depth of 6 cm or above. Due to its small seed size, the nutrients contained in the germ are not enough to support the cotyledon to emerge from the surface in the absence of light [34]. Therefore, effective weed management measures can be considered in combination with changing tillage systems and using deep tillage operations. From the results of this experiment, we can see that the seed germination rate placed on the soil surface was lower than that placed in the petri dish, and similar results were found in other studies of *Lolium rigidum* [24, 35]. This difference may be caused by poor seed-soil contact, low water utilization, lack of light, fluctuation in moisture at soil surface or possibly predation and etc [24].

The studies above have shown that weed seed germination is determined by the interaction of various factors, which are both influenced by genetics and driven by environment [36]. Other studies suggest that alleles equipped with herbicide resistance have pleiotropic impacts on the life cycle of weeds [37, 38]. The results of this study showed that in response to temperature, light, salt, pH, osmotic potential and buried depth, the germination ability of resistant species was almost all lower than that of sensitive species. The significant germination delay in the adverse environment such as strong acid and alkali was considered to be an evolutionary strategy of resistant species, and the germination characteristics showed a decline in fitness. Reduced fitness of resistant plants further slows the evolution of resistance [39]. Enhanced seed dormancy can impact management strategies for preventing or controlling herbicide resistance, including the rotation of herbicides [40]. Results from these experiments may be helpful for the design of weed management strategies, such as deep tillage, cover crop, crop rotation, etc.

## Conclusion

As a malignant weed in agricultural systems, *A*. *retroflexus* has been reported to have evolved varying degrees of resistance to the commonly used herbicide nicosulfuron. The results showed that both the resistant and sensitive populations could germinate in a wide range of osmotic potential, salt and pH values, which were not the main abiotic limiting factors for the germination of this species. The extraordinary ability of *A*. *retroflexus* to germinate under a variety of abiotic stress enables it to be a widespread and problematic weed in different areas. Our findings also demonstrated that there were variations in germination characteristics between nicosulfuron-resistant and -sensitive populations, which possibly depend on the resistance genes and their different genetic background. Germination of *A*. *retroflexus* had a strong relationship with temperature and burial depth. At lower temperature of 25°C/20°C, its germination would reduce greatly, and when the burial depth was ≥ 6 cm, both the R and S populations can not germinate. Thus, proper early planting time, optimum tillage depth and cover crop residue cover on the soil surface could be studied further for its management.

## Supporting information

S1 Dataset (XLSX)
